# Neoadjuvant and Adjuvant Chemotherapy for Variant Histology Bladder Cancers: A Systematic Review and Meta-Analysis

**DOI:** 10.3389/fonc.2022.907454

**Published:** 2022-07-14

**Authors:** Ziwei Zhu, Yunyuan Xiao, Shengye Hu, Ziyuan Wang, Zaisheng Zhu

**Affiliations:** ^1^ Department of Urology, Affiliated Jinhua Hospital, Zhejiang University School of Medicine, JinHua, China; ^2^ Key Laboratory of Laparoscopic Technique Research of Zhejiang Province, Department of General Surgery, Sir Run Run Shaw Hospital, Zhejiang University School of Medicine, Hangzhou, China

**Keywords:** urinary bladder neoplasms, meta-analysis, variant histology, neoadjuvant chemotherapy, adjuvant chemotherapy

## Abstract

**Context:**

To improve the prognosis of variant histology (VH) bladder cancers, clinicians have used neoadjuvant chemotherapy (NAC) or adjuvant chemotherapy (AC) on the basis of radical cystectomy (RC). Despite some new data, the evidence remains mixed on their efficacy.

**Objective:**

To update the current evidence on the role of NAC and AC for VH bladder cancers.

**Evidence Acquisition:**

We searched for all studies investigating NAC or AC for bladder cancer patients with variant histology in PubMed, Embase, and the Cochrane Central Register of Controlled Trials up to December 2021. The primary end points were recurrence-free survival (RFS), cancer-specific survival (CSS), and overall survival (OS).

**Evidence Synthesis:**

We identified 18 reports comprising a total of 10,192 patients in the NAC studies. In patients with VH, the use of NAC did improve CSS (hazard ratio [HR] 0.74, 95% confidence interval [CI] 0.55–0.99, p = 0.044), and OS (HR 0.74, 95% CI 0.66–0.84, p = 0.000), but not RFS (HR 1.15, 95% CI 0.56–2.33, p = 0.706). Subgroup analyses demonstrated that receiving NAC was associated with better OS in sarcomatoid VH (HR 0.67, 95% CI 0.54–0.83, p = 0.000) and neuroendocrine VH (HR 0.54, 95% CI 0.43–0.68, p = 0.000). For AC, we identified eight reports comprising a total of 3254 patients. There was a benefit in CSS (HR 0.61, 95% CI 0.43–0.87, p = 0.006) and OS (HR 0.76, 95% CI 0.60–0.98, p = 0.032). Subgroup analyses demonstrated that only neuroendocrine VH had better CSS (HR 0.29, 95% CI 0.13–0.67, p = 0.174) when receiving AC.

**Conclusions:**

NAC or AC for VH bladder cancers confers an OS and CSS benefit compared with RC alone. For NAC, the benefit was independently observed in the sarcomatoid and neuroendocrine subgroups. As for AC, only neuroendocrine subgroups improved CSS.

**Systematic Review Registration:**

https://www.crd.york.ac.uk/prospero/, identifier CRD42021289487.

## 1 Introduction

Bladder cancer is the 10th most commonly diagnosed cancer worldwide, with approximately 573,000 new cases and 213,000 deaths in 2020 (1). In patients with bladder cancer, about 75% of instances are classified as pure urothelial carcinoma, while the remaining 25% harbor variant histology (VH) (2–4). The presence of VH has been regarded as a poor prognostic factor in several studies (4–6). Compared with pure urothelial carcinoma, VH in patients with urothelial carcinoma of the bladder is associated with increased risks of disease recurrence as well as cancer-specific and overall mortality (7). However, there is a relative gap in knowledge of the treatment in these patients.

At present, whether neoadjuvant chemotherapy (NAC) or adjuvant chemotherapy (AC) is effective for VH bladder cancers is still uncertain. For example, while a secondary analysis of the Southwest Oncology Group directed intergroup randomized trial S8710 suggests NAC was an independent predictor of improved overall survival and cancer-specific survival on multivariate analysis in bladder cancer patients with squamous and glandular differentiation (8), some single-center retrospective studies concluded that the use of NAC did not improve recurrence-free, cancer-specific or overall survival (9–11). In the use of AC, there is a small case series found that no administration of AC was independently associated with poor overall survival in cases with glandular differentiation. Conversely, another study reported the administration of AC did not significantly improve survival outcomes in any histological variant (12).

Evidence concerning NAC or AC in the treatment of patients with histological variants is scarce and quite divergent. Thus, we performed this systematic review and meta-analysis to summarize the current data and to determine whether NAC or AC is effective for VH bladder cancers.

## 2 Evidence Acquisition

### 2.1. Search Strategy

This systematic review was performed according to the Preferred Reported Items for Systematic Reviews and Meta-Analyses (PRISMA) guidelines, and registered with the International Prospective Register of Systematic Reviews (CRD42021289487) (13). A systematic literature search of PubMed, Embase, and the Cochrane Central Register of Controlled Trials was performed to identify studies regarding the role of chemotherapy in VH performed prior to December 2021. The following terms were used: “urinary bladder neoplasms/transitional cell carcinoma/bladder cancer,” “variant/difference/mix,” “adjuvant chemotherapy/neoadjuvant chemotherapy,” “cystectomy,” “multivariable/adjusted,” and relevant variants of these search terms. The full search term algorithms are shown in **Appendix 1**. The literature search was unrestricted concerning publication date, region, and language. Studies that were performed at the same centers with overlapping time periods were excluded. Full articles were retrieved for further review.

### 2.2. Inclusion Criteria and Study Eligibility

The eligibility of each study was evaluated taking into account participants, interventions, comparators, outcomes, and study design approach (PICOS): Participants, bladder cancer patients with VH who intended to undergo radical cystectomy; Interventions, bladder cancer patients with VH who underwent radical cystectomy with systemic NAC or AC; Comparators, bladder cancer patients with VH who only underwent radical cystectomy; Outcomes, comparison of overall survival (OS), cancer-specific survival (CSS), and recurrence-free survival (RFS); and Study design, no restrictions on research design, but only studies with multivariate analyses were considered for meta-analysis. We considered randomized controlled trials (RCTs) and nonrandomized observational studies, as well as population-based cohorts (Surveillance, Epidemiology, and End Results [SEER], National Cancer Data Base [NCDB]) for inclusion into the systematic review and meta-analysis.

Reviews, letters, editorials, and case reports were excluded. In case of multiple reports of the same cohort, the most complete data aggregated with the longest follow-up duration were selected. In case different outcomes were examined, both articles were included to gather comprehensive data.

### 2.3. Data Extraction

Data extraction was performed by two authors (Zw Zhu and Yy Xiao) with any discrepancy resolved by a third author (Zs Zhu). Data on the paper (first author name, publication year, country, center, period of patient recruitment, and study type), participant demographics and oncologic characteristics (VH type, clinical T stage, and pathological TN stage), treatment characteristics (type of chemotherapy regimen and follow-up duration), outcomes (OS, CSS, RFS, pCR, and pDS), and results (numbers of events, hazard ratios [HRs], 95% confidence intervals [CIs], and p-values) were extracted.

### 2.4. Risk of Bias Assessment

The Cochrane Handbook for Systematic Reviews of Interventions was used to assess the risk of bias. Due to only nonrandomized comparative studies, RoB was determined by examining the risk of preassigned confounders. The confounding factors were identified as the most important prognostic factors at the time of treatment. The articles were therefore reviewed based on the adjustment for the effects of age, gender, tumor staging and grading, positive surgical margins, and receipt of NAC/AC. The RoB of each study was assessed independently by two authors (Zw Zhu and Yy xiao). The overall RoB level was judged as “low,” “intermediate,” or “high”.

### 2.5. Statistical Analyses

VH was defined as nonpure urothelial carcinoma, including urothelial carcinoma with VH or pure VH in this analysis. The effects of NAC/AC on OS, RFS, and CSS were measured using hazard ratios (HRs). In studies with only HRs and p-values we calculated the corresponding 95% CIs (14, 15). Forest plots were used to assess HRs to describe the relationships between NAC (or AC) and OS, RFS, and CSS. Subgroup analyses of “micropapillary,” “squamous,” “glandular,” “sarcomatoid,” and “small cell” VH were performed.

Between-study heterogeneity was assessed using χ² and I² tests. A Cochran Q statistic p-value < 0.05 and I² statistic >50% indicate statistically significant heterogeneity between trials (16). When no significant heterogeneity was observed, fixed-effect models through the inverse-variance method were used for calculation. In the event that at least 10 studies were included, funnel plots were to be used to assess publication bias.

Sensitivity analyses were conducted, where we removed each study one at a time, to determine the impact on the overall pooled result.

All statistical analyses were performed using Review Manager version 5.4.1(The Cochrane Collaboration, 2020.) and STATA/MP 14.0 (Stata-Corporation, 2014.).

## 3 Results

### 3.1. Study Selection and Characteristics

**Figure 1:**
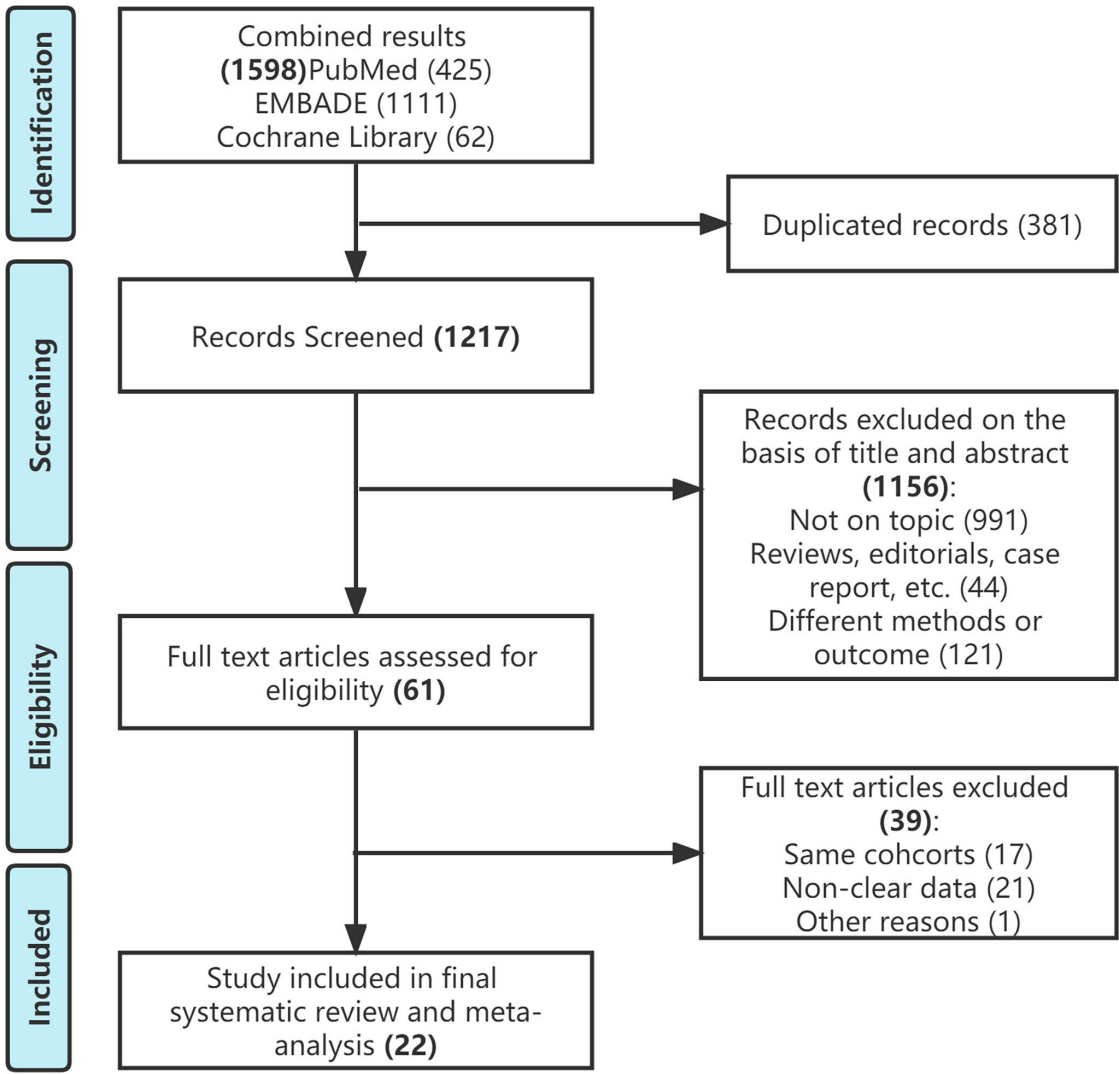
Study selection flowchart according to the Preferred Reporting Items for Systematic Reviews and Meta-Analysis guidelines.

The initial search identified 1598 publications. (425 in PubMed, 1111 in EMBASE, and 62 in Cochran library). Of these, 1217 studies remained for review after removing duplicates. A total of 1156 articles were excluded after screening the titles and abstracts, and a full text review was performed for 61 articles. After applying the selection criteria, we included 22 studies in the final analysis (**Figure 1**). All included studies were non-randomized and observational. The RoB assessment indicated an intermediate to high level of bias across the studies (**Supplementary Figure S1**).

There were 14 studies evaluating the role of NAC for VH (8, 10, 11, 17–27). Six were populations-based studies, seven studies came from different centers, and one study was based on both center and SEER. There were four studies evaluating the role of AC for VH (12, 28–30). Two were populations-based studies, while the remaining were based on different center’s database. Four studies assessed both NAC and AC in VH, three of which were based on NCDB (9, 31–33). There were a total of 18 reports comprising 10,192 participants in the NAC studies, 978 (9.6%) of which had received NAC. With the exception of six studies that ignored patients’ clinical stage, most of the studies included MIBC patients (**Supplementary Table S1**). A total of eight reports comprising 3254 people was included in the AC studies, of whom 665 received AC. Three of them included MIBC patients, the remaining five included all VH patients regardless of their stage (**Supplementary Table S2**).

### 3.2. Meta-Analysis

#### 3.2.1. Neoadjuvant and Adjuvant Chemotherapy in VH

**Figure 2:**
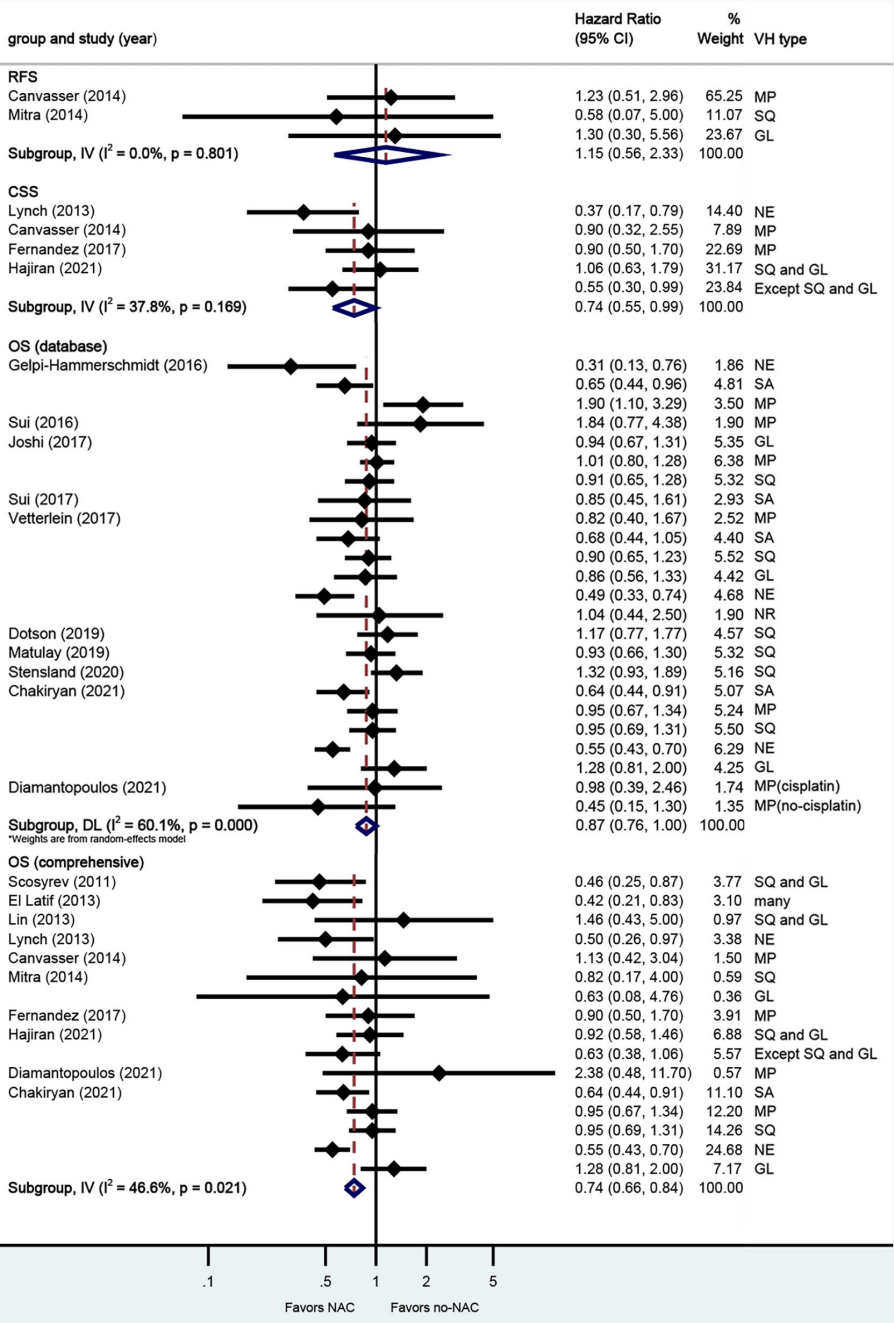
Forest plots of studies investigating the association of neoadjuvant chemotherapy with survival outcomes in variant histology. MP, micropapillary; SQ, squamous; GL, glandular; NE, neuroendocrine; SA, sarcomatoid; NR, not reported.

A total of 18 studies reported on survival outcomes in patients who had NAC prior to RC. Forest plots of HR and 95% CI for RFS, CSS, and OS are illustrated in **Figure 2**. The Cochrane Q-test (chi-square 57.60, p [0.000]) and I2 test (60.1%) revealed significant heterogeneity in OS (data base), with the funnel plot identifying five studies over the pseudo-95% CI (**Supplementary Figure S2**.). No significant heterogeneity in the Cochrane Q or I2 test was detected for other end points. Ten studies reported on the data base with a pooled HR of 0.87 (95% CI 0.76–1.00, p = 0.052) on OS. We selected the study by Chakiryan et al. for final analysis because it had high weight and stable RoB performance. Receiving NAC was not associated with RFS (HR 1.15, 95% CI 0.56–2.33, p = 0.706), but associated with better CSS (HR 0.74, 95% CI 0.55–0.99, p = 0.044) and better OS (HR 0.74, 95% CI 0.66–0.84, p = 0.000) in this pooled analysis.

**Figure 3:**
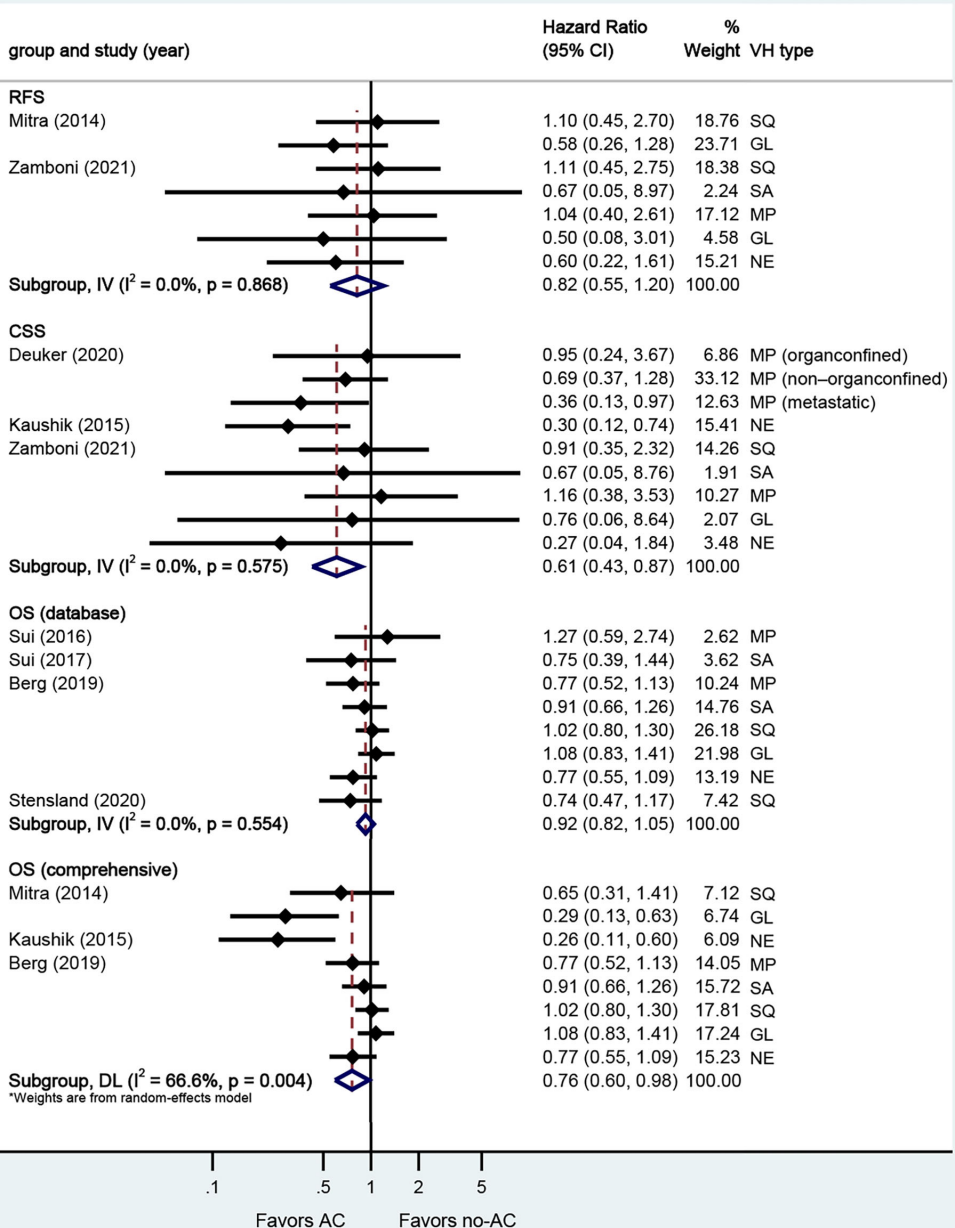
Forest plots of studies investigating the association of adjuvant chemotherapy with survival outcomes in variant histology. MP, micropapillary; SQ, squamous; GL, glandular; NE, neuroendocrine; SA, sarcomatoid; NR, not reported.

A total of eight studies reported on survival outcomes in patients who had AC after RC. Forest plots of HR and 95% CI for RFS, CSS, and OS are illustrated in **Figure 3**. The Cochrane Q-test (chi-square 20.95, p [0.004]) and I2 test (66.6%) revealed significant heterogeneity in OS (comprehensive), with the funnel plot identifying two studies over the pseudo-95% CI (**Supplementary Figure S3**). No significant heterogeneity in the Cochrane Q or I2 test was detected for other end points. Four studies reported on the data base with a pooled HR of 0.92 (95% CI 0.82–1.05, p = 0.209) on OS. We selected the study by Berg et al. for final analysis because it had high weight and stable RoB performance. Receiving AC was not associated with RFS (HR 0.82, 95% CI 0.55–1.20, p = 0.304), but associated with better CSS (HR 0.61, 95% CI 0.43–0.87, p = 0.006), and better OS (HR 0.76, 95% CI 0.60–0.98, p = 0.032) in this pooled analysis.

#### 3.2.2. Neoadjuvant and Adjuvant Chemotherapy in Micropapillary VH

**Figure 4:**
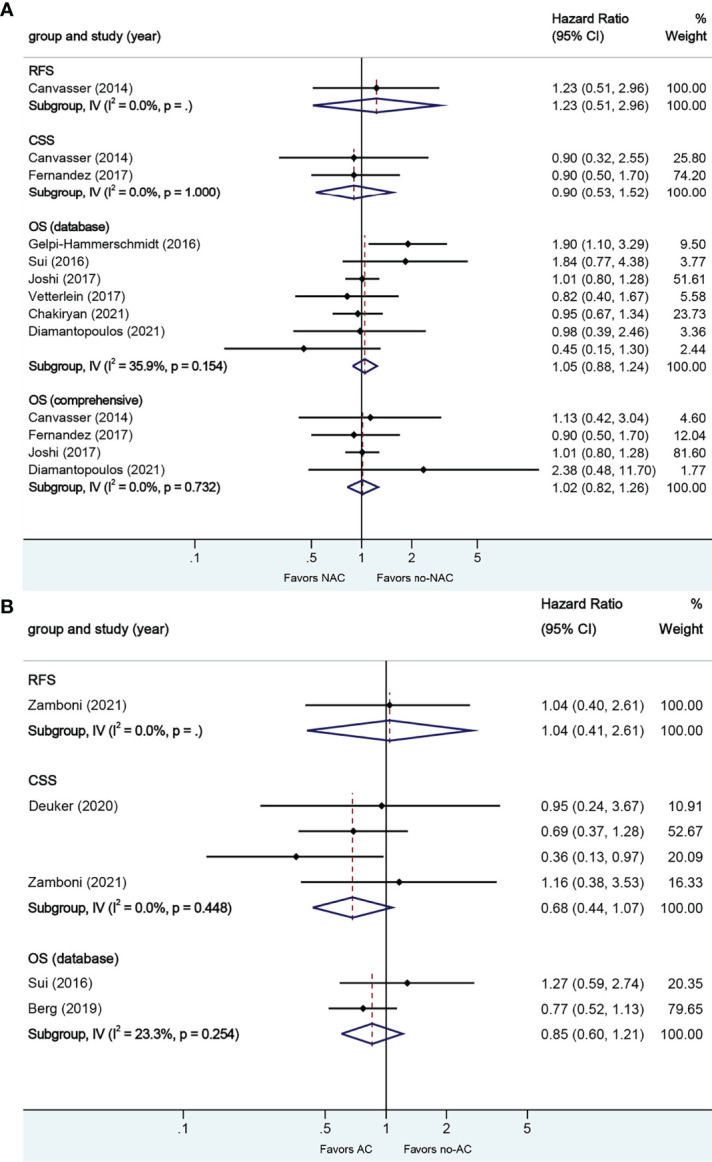
Forest plots of studies investigating the association of chemotherapy with survival outcomes in micropapillary variant histology. **(A)** neoadjuvant chemotherapy; **(B)** adjuvant chemotherapy.

A total of eight studies reported on survival outcomes in patients who had NAC prior to RC. Forest plots of HR and 95% CI for RFS, CSS, and OS are illustrated in **Figure 4A**. No significant heterogeneity in the Cochrane Q or I2 test was detected for all end points. Six studies reported on the data base with a pooled HR of 1.05 (95% CI 0.88–1.24, p = 0.597) on OS. We selected the study by Joshi et al. for final analysis because it had high weight and stable RoB performance. Receiving NAC was not associated with RFS (HR 1.23, 95% CI 0.51–2.96, p = 0.644), CSS (HR 0.90, 95% CI 0.53–1.52, p = 0.695), or OS (HR 1.02, 95% CI 0.82–1.26, p = 0.880) in this pooled analysis.

A total of four studies reported on survival outcomes in patients who had AC after RC. Forest plots of HR and 95% CI for RFS, CSS, and OS are illustrated in **Figure 4B**. No significant heterogeneity in the Cochrane Q or I2 test was detected for all end points. Two studies reported on the data base with a pooled HR of 0.85 (95% CI 0.60–1.21, p = 0.367) on OS. Receiving AC was not associated with RFS (HR 1.04, 95% CI 0.40–2.61, p = 0.935), CSS (HR 0.68, 95% CI 0.44–1.07, p = 0.096), or OS in this pooled analysis.

#### 3.2.3. Neoadjuvant and Adjuvant Chemotherapy in Squamous VH

**Figure 5:**
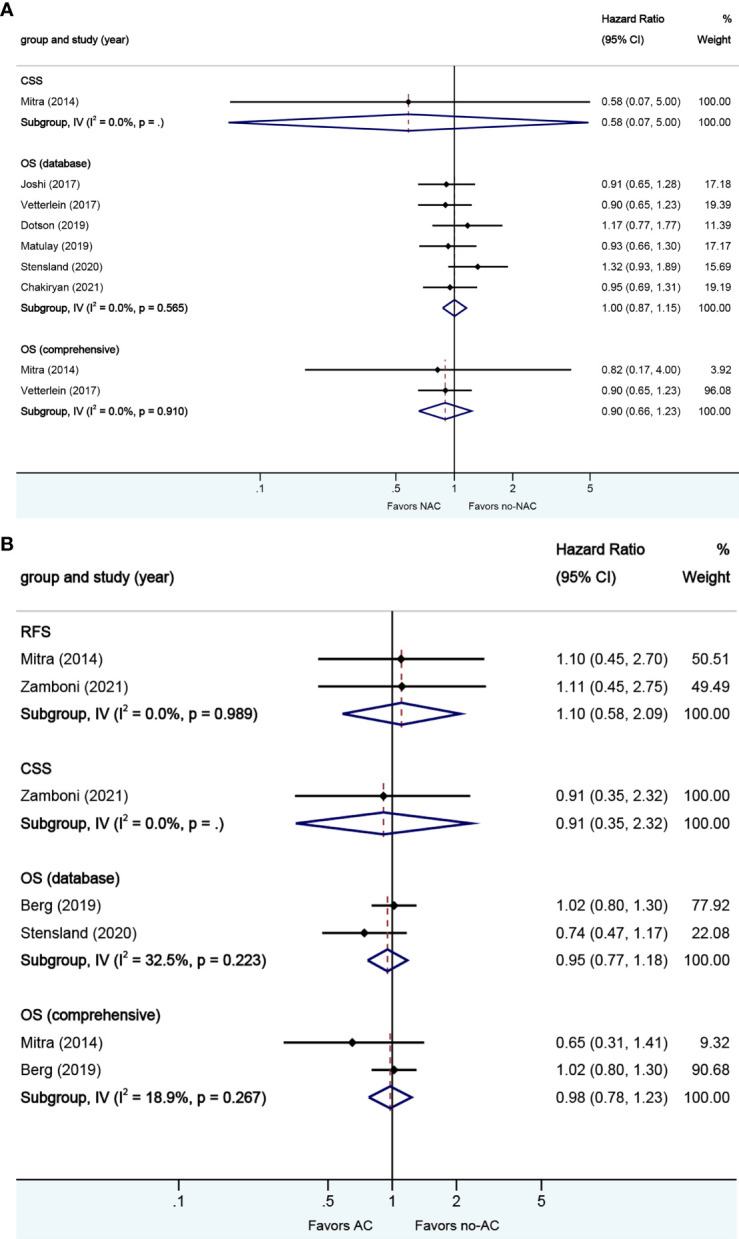
Forest plots of studies investigating the association of chemotherapy with survival outcomes in squamous variant histology. **(A)** neoadjuvant chemotherapy; **(B)** adjuvant chemotherapy.

A total of seven studies reported on survival outcomes in patients who had NAC prior to RC. Forest plots of HR and 95% CI for CSS and OS are illustrated in **Figure 5A**. No significant heterogeneity in the Cochrane Q or I2 test was detected for all end points. Six studies reported on the data base with a pooled HR of 1.00 (95% CI 0.87–1.15, p = 0.972) on OS. We selected the study by Vetterlein et al. for final analysis because it had high weight and stable RoB performance. Receiving NAC was not associated with CSS (HR 0.58, 95% CI 0.07–5.00, p = 0.617) or OS (HR 0.90, 95% CI 0.66–1.23, p = 0.494) in this pooled analysis.

A total of four studies reported on survival outcomes in patients who had AC after RC. Forest plots of HR and 95% CI for RFS, CSS, and OS are illustrated in **Figure 5B**. No significant heterogeneity in the Cochrane Q or I2 test was detected for all end points. Two studies reported on the data base with a pooled HR of 0.95 (95% CI 0.77–1.18, p = 0.640) on OS. We selected the study by Berg et al. for final analysis because it had high weight and stable RoB performance. Receiving AC was not associated with RFS (HR 1.10, 95% CI 0.58–2.09, p = 0.759), CSS (HR 0.91, 95% CI 0.35–2.32, p = 0.845), or OS (HR 0.98, 95% CI 0.78–1.23, p = 0.851) in this pooled analysis.

#### 3.2.4. Neoadjuvant and Adjuvant Chemotherapy in Glandular VH

**Figure 6:**
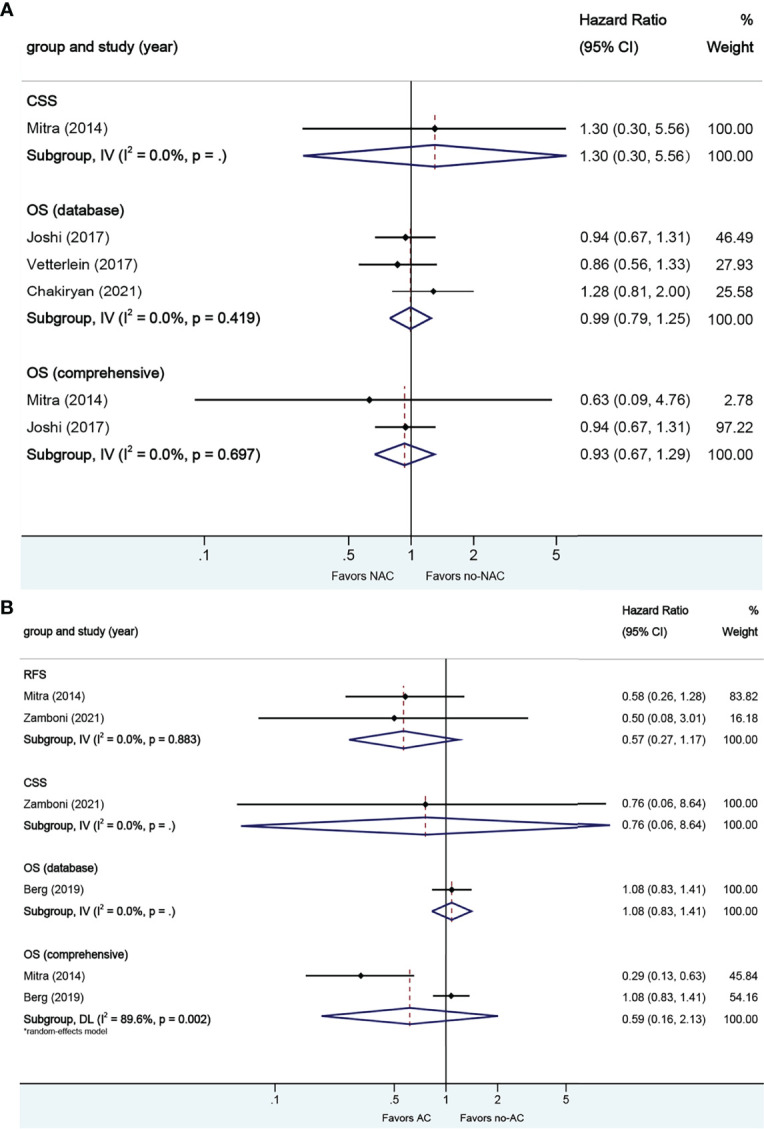
Forest plots of studies investigating the association of chemotherapy with survival outcomes in glandular variant histology. **(A)** neoadjuvant chemotherapy; **(B)** adjuvant chemotherapy.

A total of four studies reported on survival outcomes in patients who had NAC prior to RC. Forest plots of HR and 95% CI for CSS and OS are illustrated in **Figure 6A**. No significant heterogeneity in the Cochrane Q or I2 test was detected for all end points. Three studies reported on the data base with a pooled HR of 0.99 (95% CI 0.79–1.25, p = 0.972) on OS. We selected the study by Joshi et al. for final analysis because it had high weight and stable RoB performance. Receiving NAC was not associated with CSS (HR 1.30, 95% CI 0.30–5.56, p = 0.725) or OS (HR 0.93, 95% CI 0.67–1.29, p = 0.665) in this pooled analysis.

A total of three studies reported on survival outcomes in patients who had AC after RC. Forest plots of HR and 95% CI for RFS, CSS, and OS are illustrated in **Figure 6B**. The Cochrane Q-test (chi-square 9.58, p [0.002]) and I2 test (89.6%) revealed significant heterogeneity in OS (comprehensive); no significant heterogeneity in the Cochrane Q or I2 test was detected for other end points. One study based on the data base (HR 1.08, 95% CI 0.83–1.41, p = 0.569) was included in final analysis. Receiving AC was not associated with RFS (HR 0.57, 95% CI 0.27–1.17, p = 0.127), CSS (HR 0.76, 95% CI 0.06–8.64, p = 0.829), or OS (HR 0.59, 95% CI 0.16–2.14, p = 0.422) in this pooled analysis.

#### 3.2.5. Neoadjuvant and Adjuvant Chemotherapy in Sarcomatoid VH

**Figure 7:**
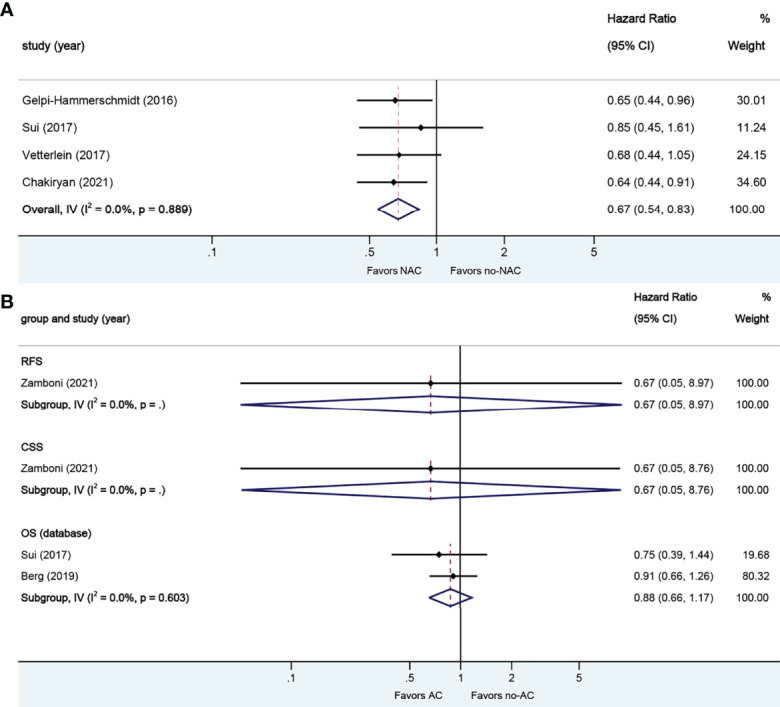
Forest plots of studies investigating the association of chemotherapy with survival outcomes in sarcomatoid variant histology. **(A)** neoadjuvant chemotherapy; **(B)** adjuvant chemotherapy.

A total of four studies reported on survival outcomes in patients who had NAC prior to RC. All of the studies reported on the data base on OS. Forest plot of HR and 95% CI for OS is illustrated in **Figure 7A**. No significant heterogeneity in the Cochrane Q or I2 test was detected for end points. Receiving NAC was associated with better OS (HR 0.67, 95% CI 0.54–0.83, p = 0.000) in this pooled analysis.

A total of three studies reported on survival outcomes in patients who had AC after RC. Forest plots of HR and 95% CI for RFS, CSS, and OS are illustrated in **Figure 7B**. No significant heterogeneity in the Cochrane Q or I2 test was detected for all end points. Two studies reported on the data base with a pooled HR of 0.88 (95% CI 0.66–1.17, p = 0.371) on OS. Receiving AC was not associated with RFS (HR 0.67, 95% CI 0.05–8.97, p = 0.762), CSS (HR 0.67, 95% CI 0.05–8.76, p = 0.761), or OS in this pooled analysis.

#### 3.2.6. Neoadjuvant and Adjuvant Chemotherapy in Neuroendocrine VH

**Figure 8:**
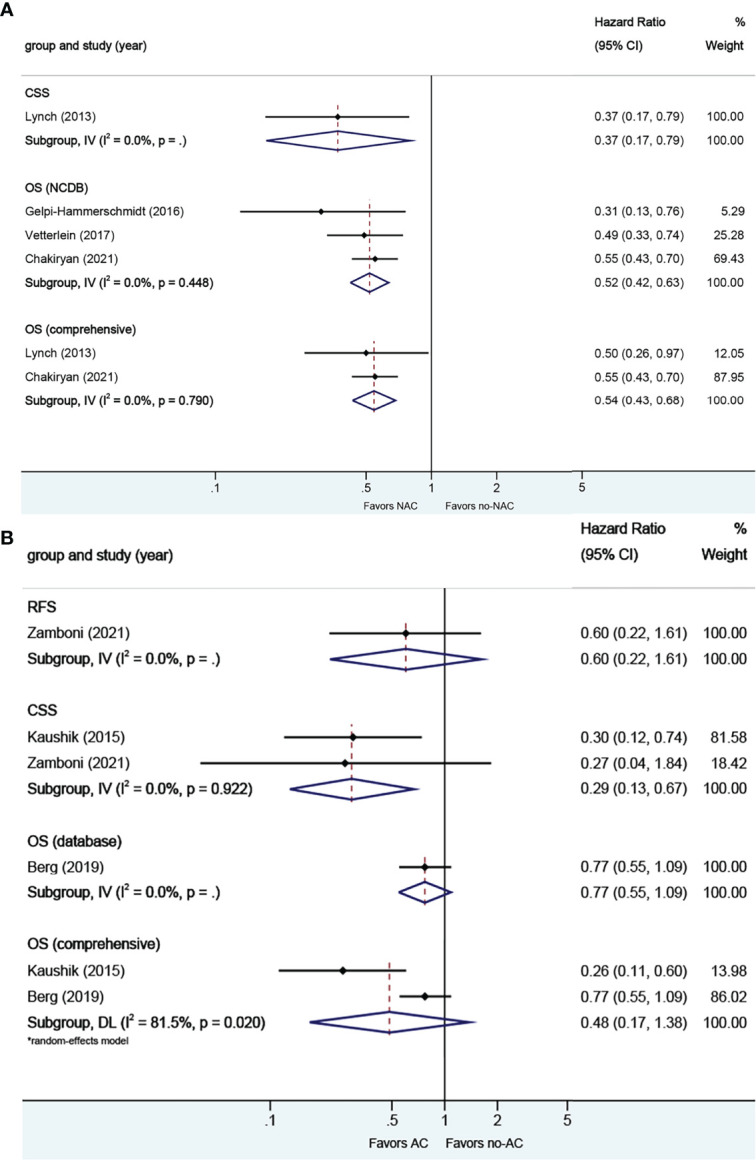
Forest plots of studies investigating the association of chemotherapy with survival outcomes in neuroendocrine variant histology. **(A)** neoadjuvant chemotherapy; **(B)** adjuvant chemotherapy.

A total of four studies reported on survival outcomes in patients who had NAC prior to RC. Forest plots of HR and 95% CI for CSS and OS are illustrated in **Figure 8A**. No significant heterogeneity in the Cochrane Q or I2 test was detected for all end points. Three studies reported on the data base with a pooled HR of 0.52 (95% CI 0.42–0.63, p = 0.000) on OS. We selected the study by Chakiryan et al. for final analysis because it had high weight and stable RoB performance. Receiving NAC was associated with better CSS (HR 0.37, 95% CI 0.17–0.79, p = 0.011) and better OS (HR 0.54, 95% CI 0.43–0.68, p = 0.000) in this pooled analysis.

A total of three studies reported on survival outcomes in patients who had AC after RC. Forest plots of HR and 95% CI for RFS, CSS, and OS are illustrated in **Figure 8B**. The Cochrane Q-test (chi-square 5.41, p [0.020]) and I2 test (81.5%) revealed significant heterogeneity in OS (comprehensive); no significant heterogeneity in the Cochrane Q or I2 test was detected for other end points. One study based on the data base (HR 0.77, 95% CI 0.55–1.09, p = 0.134) was included in final analysis. Receiving AC was not associated with RFS (HR 0.60, 95% CI 0.22–1.61, p = 0.314) or OS (HR 0.48, 95% CI 0.17–1.38, p = 0.004), but was associated with better CSS (HR 0.29, 95% CI 0.13–0.67, p = 0.174) in this pooled analysis.

### 3.3. Sensitivity Analysis

Sensitivity analyses were performed through sequential deletion of any individual study to measure the effects of each study. Overall HRs were not significantly influenced by any individual study, suggesting the robustness and reliability of the results in our meta-analysis.

## 4 Discussion

### 4.1. Role of NAC

This meta-analysis investigated the role of NAC and AC in VH bladder cancers. VH is usually associated with advanced stage, lymphovascular invasion, and lymph node metastasis (34). Although many studies have reported that patients with VH had a poor response to NAC compared to patients with pure urothelial carcinoma (35–37),the study on whether NAC is effective for VH is rare and draws different conclusions. In this respect, the present study helped identify the effect of NAC and AC in VH. Our meta-analysis demonstrated that patients with sarcomatoid and neuroendocrine VH would benefit from NAC. For patients with sarcomatoid differentiation, our findings do not align with what was envisioned. Because it is known that this rare VH is generally more aggressive and is at a more advanced stage at the time of diagnosis (38), and almost half agree immediate RC in EAU-ESMO Consensus Statements (no consensus achieved) (39).Given that the result was drawn from four populations-based studies, it should be treated with caution. In our study, no benefit was found in patients with micropapillary, squamous, or glandular VH, but we have reservations about the use of neoadjuvant chemotherapy in these patients. Although our study found no significant benefit in OS in these patients, the complete response rate (down-staging to T0) and/or partial response rate (pathological down-staging) of patients receiving NAC were obvious in some of the studies (40, 41). For example, MEEKS et al. collected data on patients with bladder cancer treated at the Memorial Sloan-Kettering Cancer Center (MSKCC) (40). Of the 44 patients with muscle-invasive micropapillary carcinoma, 29 received NAC. Down-staging to pT0 occurred in 13 (45%) of those who received neoadjuvant chemotherapy compared with two (13%) of those who did not (P = 0.049). They concluded that patients with the micropapillary variant of urothelial carcinoma should not be excluded from consideration for neoadjuvant chemotherapy. Considering the disadvantages of NAC, such as delaying RC leading to inadvertent disease progression and toxicities related to chemotherapy, administration of NAC should be used with caution.

### 4.2. Role of AC

The major downside of post-operative chemotherapy is that patients often suffer a decline in their physical performance after RC, which leads to patients being unable to tolerate chemotherapy. After RC, clinicians obtain the most accurate pathological staging from specimens. They can judge the next treatment measures *via* RC specimens. In this systematic review and meta-analysis, we found that only patients with neuroendocrine VH who received AC have a CSS benefit compared with those who underwent RC alone.

### 4.3. VH in the Future

With the development of molecular medical research, a variety of potential biomarkers have been evaluated to predict response to cisplatin-based chemotherapy (42). However, although the use of NAC/AC may be guided by tumor molecular characteristics in the future, the VH will continue to influence clinicians’ decision-making for a long time.

Based on the current study, further clinical risk stratification in patients with VH may better guide the treatment. On the basis of VH classification, Rosiello et al. further divided patients with squamous VH into three groups according to TNM stages (1:T3–4aN0M0, 2:TanyN1–3M0, 3:T4bN0–3 or M1) (29). They found that chemotherapy benefited patients in T4bN0–3 or M1, while no significant benefit was found in the 1 and 2 groups. In our meta-analysis, receiving NAC was not associated with CSS or OS in squamous VH. Deuker et al. have stratified micropapillary patients into three groups: T1-2 N0M0, T3–4 N0M0/TanyN1–3 M0, Tany Nany M1 (43). They found that chemotherapy for micropapillary VH is effective in Tany Nany M1 stages, but of no beneficial effect in the T1-2 N0M0 stage. In our meta-analysis, receiving NAC was not associated with RFS, CSS, or OS in micropapillary VH. In addition, Speir et al. divided patients with squamous VH into two cohorts based on the percentage of squamous VH in the TUR specimen: <50% or ≥50% squamous VH (44). They found favorable results in patients with <50% involvement by squamous VH who received NAC.

### 4.4. Study Strengths and Limitations

The treatment of histological variants of bladder neoplasm is strongly debated, and the role of chemotherapy has not yet been properly assessed. We performed a precise evaluation of the available studies. This review can certainly stimulate the production of randomized studies. In addition, during the search, we found a systematic review of similar topic (45). Their research focused on the systematic review and extensively introduced the study in the use of NAC for variant histologies. Compared to their study, our study focused on the meta-analysis, and the retrieval database was relatively broad, but only the studies of multivariate analysis were considered. Therefore, some of the articles included in the study are different from theirs. We believe that our conclusions are based on meta-analysis and are more reliable.

Our study is not devoid of limitations. All studies included in this meta-analysis were retrospective and might show selection bias. Furthermore, multiple series with negative results may be unpublished, and the published studies contain some small sample size research which may impact the overall quality of data. Lots of studies depended on database entries and might have suffered from a lack of secondary pathology reviews. Some studies included for analysis did not contain follow-up data or contained follow-up of less than 2 years, thus some conclusions regarding survival outcomes are unreliable.

In addition, heterogeneity was detected in the OS (AC, comprehensive) analysis, limiting the value of these results. The analyses conducted without regard to their particular VH type, and the inclusion of the populations-based studies which always account for high weight, may have contributed in large measure to significant heterogeneity in this meta-analysis. While this may be mitigated with a random-effect model, conclusions should be interpreted with caution.

Furthermore, our study investigates NAC and AC for all of VH, which may be oversimplified because not all VH types have similar biological behavior. For example, squamous VH has been considered a chemoresistant tumor, while neuroendocrine VH has been seen as sensitive to chemotherapy.

## 5 Conclusions

In conclusion, our meta-analysis found favorable OS and CSS in patients with VH who received NAC or AC. In the subgroup analyses, NAC independently improved OS in sarcomatoid and neuroendocrine subgroups. The results in AC report a significant CSS benefit in patients with neuroendocrine VH. However, this finding should be interpreted with caution because of the limitations of this studies which include the heterogeneity of the population of interest and the retrospective nature of the primary data evaluated.

## Data Availability Statement

The original contributions presented in the study are included in the article/**Supplementary Material**. Further inquiries can be directed to the corresponding author.

## Author Contributions

ZWZ, ZW, and ZSZ put forward the concept of the study and designed the study. SH and YX contributed to the data acquisition. ZWZ and ZW contributed to prepare the manuscript and the statistical analysis. ZSZ reviewed the manuscript. All authors read and approved the final manuscript.

## Conflict of Interest

The authors declare that the research was conducted in the absence of any commercial or financial relationships that could be construed as a potential conflict of interest.

## Publisher’s Note

All claims expressed in this article are solely those of the authors and do not necessarily represent those of their affiliated organizations, or those of the publisher, the editors and the reviewers. Any product that may be evaluated in this article, or claim that may be made by its manufacturer, is not guaranteed or endorsed by the publisher.
