# Supplementary material for: Neoadjuvant and Adjuvant Chemotherapy for Variant Histology Bladder Cancers: A Systematic Review and Meta-Analysis
Source: Front Oncol. 2022 Jul 14;12:907454. doi: 10.3389/fonc.2022.907454 (PMC9333064; doi:10.3389/fonc.2022.907454)
Supplement: Supplementary file 1 [file DataSheet_1.pdf]

NAC:

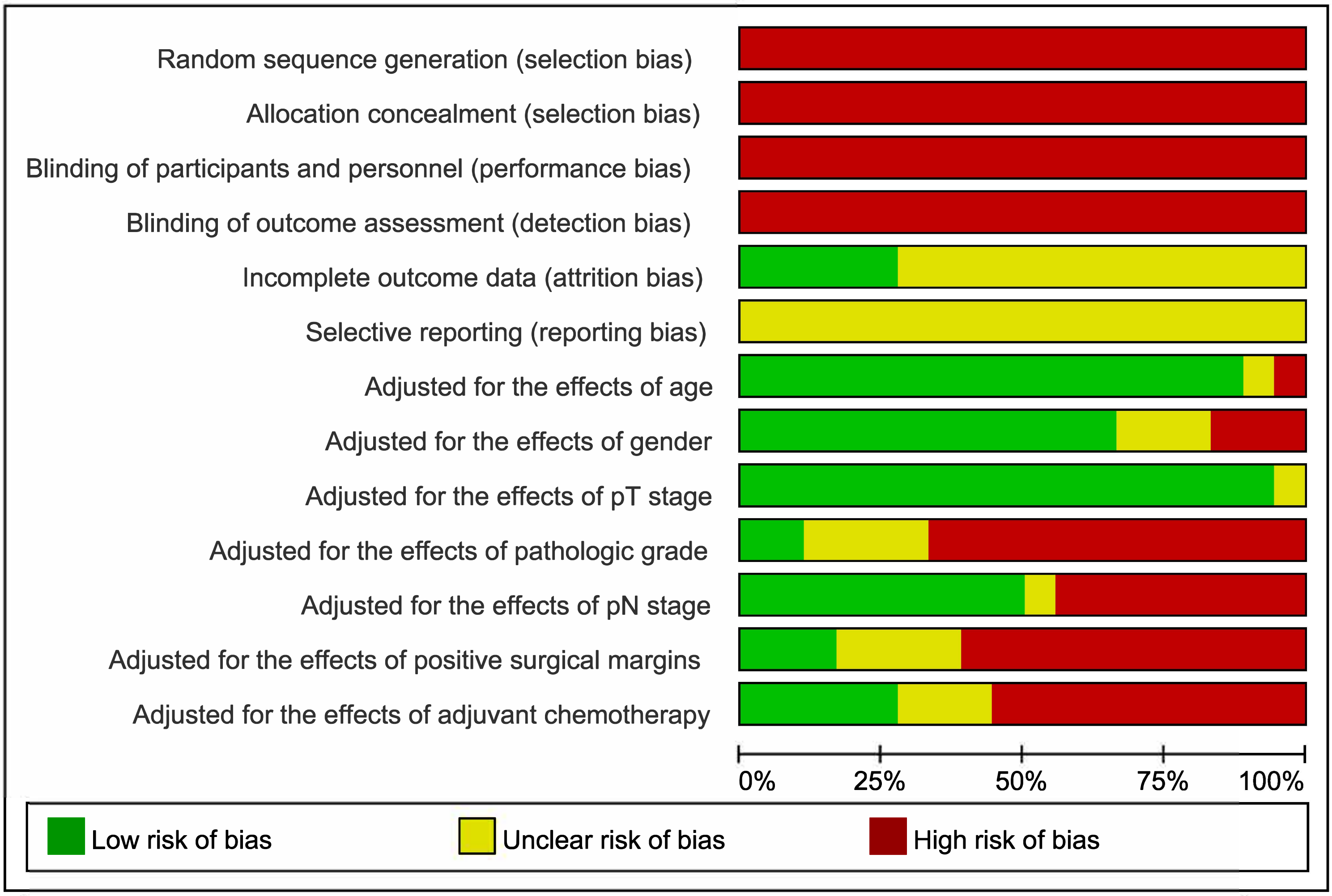
Random sequence generation (selection bias)
Allocation concealment (selection bias)
Blinding of participants and personnel (performance bias)
Blinding of outcome assessment (detection bias)
Incomplete outcome data (attrition bias)
Selective reporting (reporting bias)
Adjusted for the effects of age
Adjusted for the effects of gender
Adjusted for the effects of pT stage
Adjusted for the effects of pathologic grade
Adjusted for the effects of pN stage
Adjusted for the effects of positive surgical margins
Adjusted for the effects of adjuvant chemotherapy
0%
25%
50%
75%
100%
Low risk of bias
Unclear risk of bias
High risk of bias

	Random sequence generation (selection bias)	Allocation concealment (selection bias)	Blinding of participants and personnel (performance bias)	Blinding of outcome assessment (detection bias)	Incomplete outcome data (attrition bias)	Selective reporting (reporting bias)	Adjusted for the effects of age	Adjusted for the effects of gender	Adjusted for the effects of pT stage	Adjusted for the effects of pathologic grade	Adjusted for the effects of pN stage	Adjusted for the effects of positive surgical margins	Adjusted for the effects of adjuvant chemotherapy
Canvasser 2014	−	−	−	−	?	?	+	+	+	−	−	−	−
Chakiryan 2021	−	−	−	−	+	?	+	+	+	−	+	−	−
Diamantopoulos 2021	−	−	−	−	?	?	+	+	+	−	+	+	−
Dotson 2019	−	−	−	−	?	?	+	−	+	−	−	−	−
El Latif 2013	−	−	−	−	?	?	+	+	+	−	+	+	−
Fernandez 2017	−	−	−	−	?	?	−	−	+	−	−	−	−
Gelpi-Hammerschmidt 2016	−	−	−	−	?	?	?	?	?	?	?	?	?
Hajiran 2021	−	−	−	−	?	?	+	?	+	?	+	?	?
Joshi 2017	−	−	−	−	?	?	+	+	+	?	+	?	?
Lin 2013	−	−	−	−	?	?	+	+	+	−	−	+	−
Lynch 2013	−	−	−	−	+	?	+	+	+	−	−	−	−
Matulay 2019	−	−	−	−	?	?	+	+	+	+	−	−	−
Mitra 2014	−	−	−	−	?	?	+	?	+	?	+	?	+
Scosyrev 2011	−	−	−	−	+	?	+	−	+	−	+	−	−
Stensland 2020	−	−	−	−	+	?	+	+	+	−	+	−	+
Sui 2016	−	−	−	−	?	?	+	+	+	+	−	−	+
Sui 2017	−	−	−	−	?	?	+	+	+	−	−	−	+
Vetterlein 2017	−	−	−	−	+	?	+	+	+	−	+	−	+

AC:

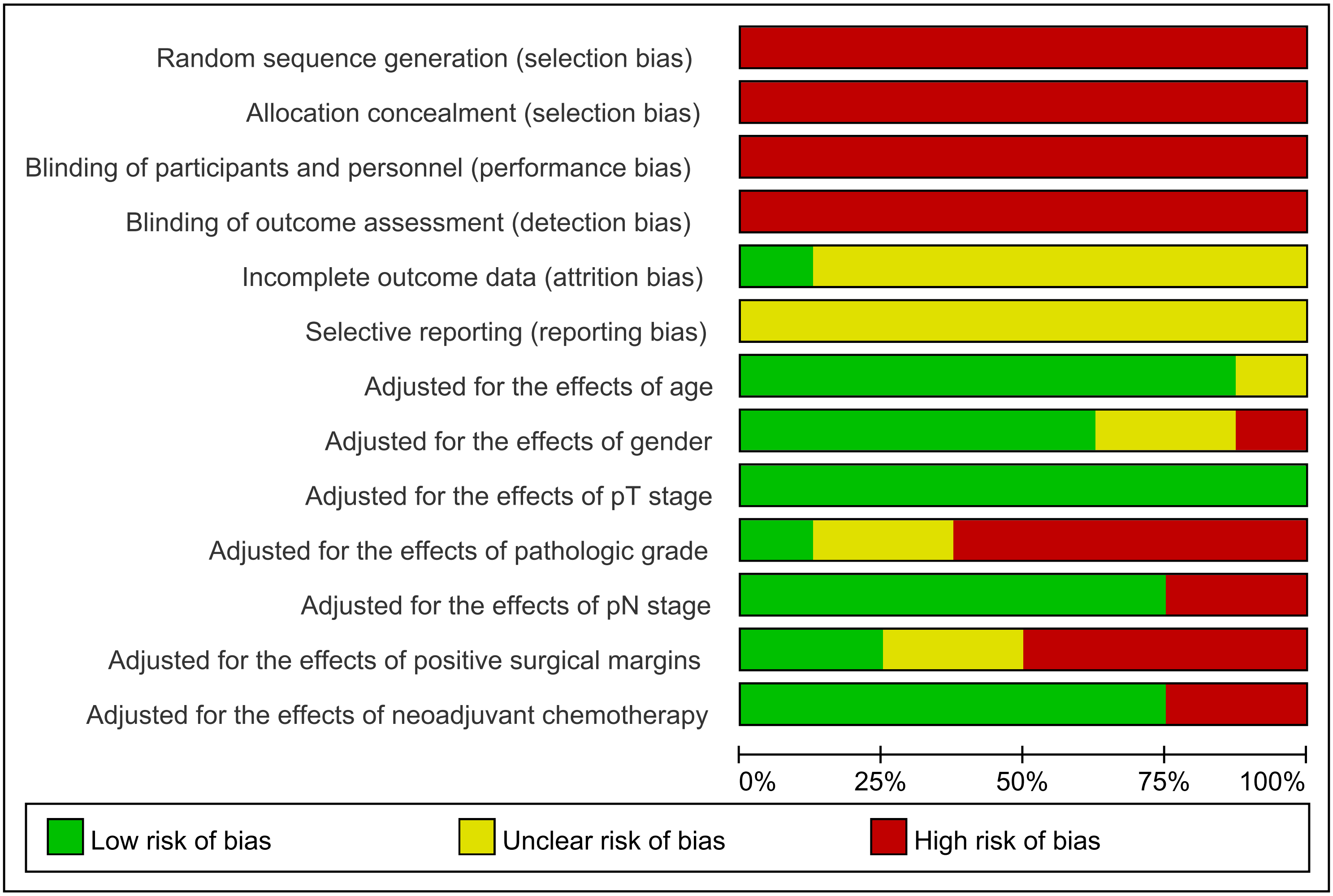

Random sequence generation (selection bias)
Allocation concealment (selection bias)
Blinding of participants and personnel (performance bias)
Blinding of outcome assessment (detection bias)
Incomplete outcome data (attrition bias)
Selective reporting (reporting bias)
Adjusted for the effects of age
Adjusted for the effects of gender
Adjusted for the effects of pT stage
Adjusted for the effects of pathologic grade
Adjusted for the effects of pN stage
Adjusted for the effects of positive surgical margins
Adjusted for the effects of neoadjuvant chemotherapy
0%
25%
50%
75%
100%
Low risk of bias
Unclear risk of bias
High risk of bias

	Random sequence generation (selection bias)	Allocation concealment (selection bias)	Blinding of participants and personnel (performance bias)	Blinding of outcome assessment (detection bias)	Incomplete outcome data (attrition bias)	Selective reporting (reporting bias)	Adjusted for the effects of age	Adjusted for the effects of gender	Adjusted for the effects of pT stage	Adjusted for the effects of pathologic grade	Adjusted for the effects of pN stage	Adjusted for the effects of positive surgical margins	Adjusted for the effects of neoadjuvant chemotherapy
Berg 2019	−	−	−	−	?	?	+	+	+	−	+	+	−
Deuker 2020	−	−	−	−	?	?	+	−	+	−	+	−	−
Kaushik 2015	−	−	−	−	?	?	?	?	+	?	+	?	+
Mitra 2014	−	−	−	−	?	?	+	?	+	?	+	?	+
Stensland 2020	−	−	−	−	+	?	+	+	+	−	+	−	+
Sui 2016	−	−	−	−	?	?	+	+	+	+	−	−	+
Sui 2017	−	−	−	−	?	?	+	+	+	−	−	−	+
Zamboni 2021	−	−	−	−	?	?	+	+	+	−	+	+	+
