# Supplementary figures and images for: Neoadjuvant and Adjuvant Chemotherapy for Variant Histology Bladder Cancers: A Systematic Review and Meta-Analysis
Source: Front Oncol. 2022 Jul 14;12:907454. doi: 10.3389/fonc.2022.907454 (PMC9333064; doi:10.3389/fonc.2022.907454)

Funnel plot with pseudo 95% confidence limits

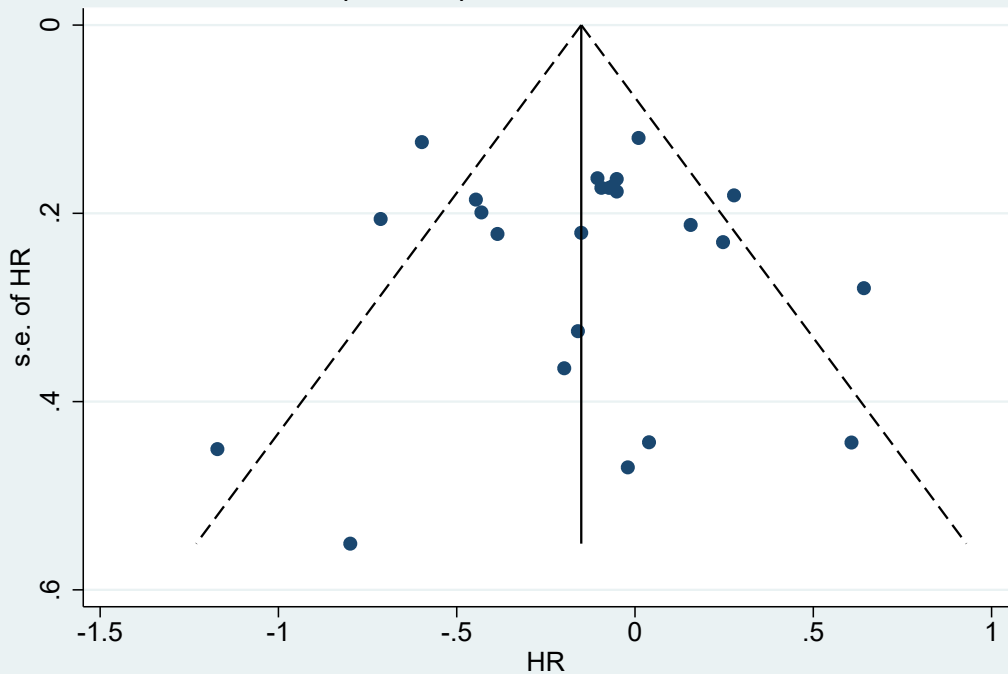

Supplement: Supplementary file 2 [file DataSheet_2.pdf]

Funnel plot with pseudo 95% confidence limits

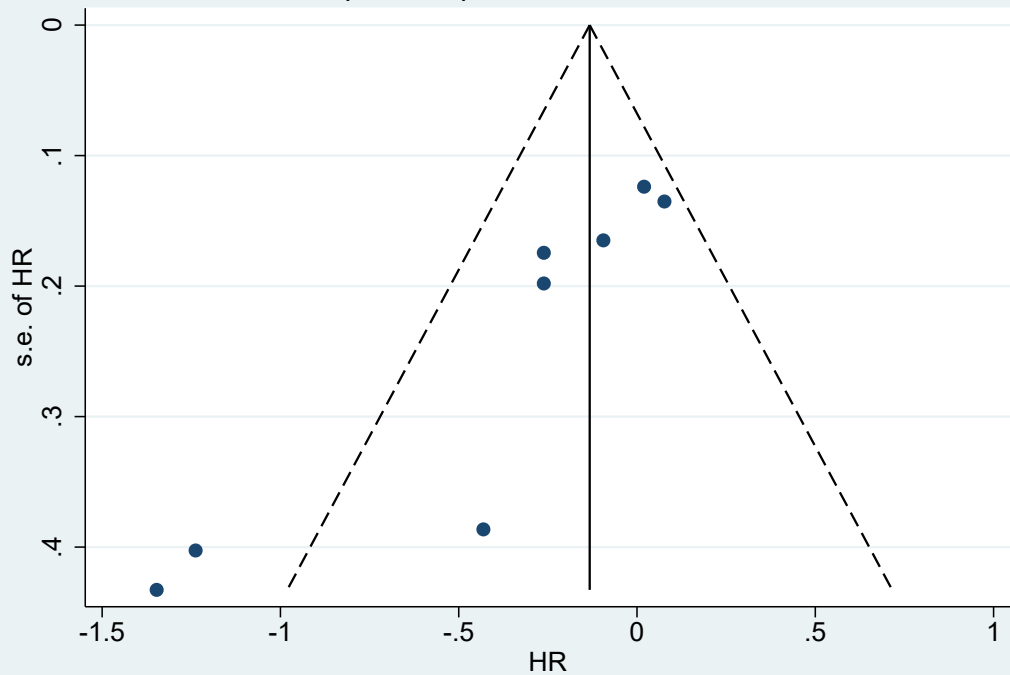

Supplement: Supplementary file 3 [file DataSheet_3.pdf]
