# Supplementary material for: Neoadjuvant and Adjuvant Chemotherapy for Variant Histology Bladder Cancers: A Systematic Review and Meta-Analysis
Source: Front Oncol. 2022 Jul 14;12:907454. doi: 10.3389/fonc.2022.907454 (PMC9333064; doi:10.3389/fonc.2022.907454)
Supplement: Supplementary file 4 [file DataSheet_4.doc]

**search syntax and search string**

Database: Pubmed < to 2021 Dec 01 > 425 Search Strategy:

1. (((((cancer*[Title/Abstract]) OR (carcinom*[Title/Abstract])) OR (tumor*[Title/Abstract])) OR (papillom*[Title/Abstract])) OR (neoplasm*[Title/Abstract])) AND ((urothel*[Title/Abstract]) OR (bladder[Title/Abstract]) OR (Transitional Cell[Title/Abstract]))
2. "Carcinoma, Transitional Cell"[Mesh]
3. "Urinary Bladder Neoplasms"[Mesh]
4. 1 or 2 or 3
5. (nonpure[Title/Abstract]) OR (variant*[Title/Abstract])
6. (difference*[Title/Abstract]) OR (differentiation*[Title/Abstract])
7. (MIX[Title/Abstract]) OR (mixed[Title/Abstract]) OR (mixes[Title/Abstract])
8. ("Carcinoma, Squamous Cell"[Mesh]) OR (Squamous[Title/Abstract])
9. ("Adenocarcinoma"[Mesh]) OR (Adenocarcinom*[Title/Abstract])
10. ("Carcinoma, Small Cell"[Mesh]) OR (Small Cell[Title/Abstract])
11. Micropapillary[Title/Abstract]
12. Sarcomatoid[Title/Abstract]
13. Lymphoepithelioma-Like[Title/Abstract]
14. or/5-13
15. 4 and 14
16. "Neoadjuvant Therapy"[Mesh]
17. "Antineoplastic Agents"[Mesh]
18. "Drug Therapy"[Mesh]
19. "drug therapy" [Subheading]
20. "Drug Therapy, Combination"[Mesh]
21. chemo*[Title/Abstract]
22. ("Epirubicin"[Mesh]) OR (epirubicin)
23. ("Doxorubicin"[Mesh]) OR (Doxorubicin)
24. Adriamycin[Title/Abstract]
25. gemcitabine[Title/Abstract]
26. ("Mitomycins"[Mesh]) OR (Mitomycin)
27. ("Thiotepa"[Mesh]) OR (Thiotepa)
28. or/16-27
29. 15 and 28
30. "Cystectomy"[Mesh]
31. (cystectom*[Title/Abstract])
32. 30 OR 31
33. 29 and 32
34. "Multivariate Analysis"[Mesh]
35. "Risk Assessment"[Mesh]
36. "Kaplan-Meier Estimate"[Mesh]
37. (Multivaria*[Title/Abstract])
38. (adjusted[Title/Abstract])
39. (Cox[Title/Abstract])
40. or/34-39
41. 33 and 40

Database: Embase < to 2021 Dec 01 > 1,111 Search Strategy:

#69. #59 AND #68

#68. #60 OR #61 OR #62 OR #63 OR #64 OR #65 OR #66 OR #67

#67. 'adjusted hazard ratio'/exp

#66. 'adjusted':ab,kw,ti

#65. 'kaplan meier':ab,kw,ti

#64. 'cox':ab,kw,ti

#63. 'multivaria*':ab,kw,ti

#62. 'risk assessment'/exp

#61. 'multivariate analysis'/exp

#60. 'kaplan meier method'/exp

#59. #55 AND #58

#58. #56 OR #57

#57. 'cystectom*':ab,kw,ti

#56. 'cystectomy'/exp

#55. #29 AND #54

#54. #30 OR #31 OR #32 OR #33 OR #34 OR #35 OR #36 OR #37 OR #38 OR #39 OR #40 OR #41 OR #42 OR #43 OR #44 OR #45 OR #46 OR #47 OR #48 OR #49 OR #50 OR #51 OR #52 OR #53

#53. 'drug therapy'/exp

#52. 'thio-tepa':ab,kw,ti

#51. 'thiotepa':ab,kw,ti

#50. 'gemcitabine':ab,kw,ti

#49. 'adriamycin':ab,kw,ti

#48. 'mitomycin*':ab,kw,ti

#47. 'doxorubicin':ab,kw,ti

#46. 'epirubicin':ab,kw,ti

#45. 'drug therapy':ab,kw,ti

#44. 'chemo*':ab,kw,ti

#43. 'thiotepa'/exp

#42. 'gemcitabine'/exp

#41. 'mitomycin derivative'/exp

#40. 'mitomycin'/exp

#39. 'doxorubicin derivative'/exp

#38. 'doxorubicin'/exp

#37. 'epirubicin'/exp

#36. 'neoadjuvant therapy'/exp

#35. 'antineoplastic antibiotic'/exp

#34. 'antineoplastic agent'/exp

#33. 'cancer combination chemotherapy'/exp

#32. 'combination chemotherapy'/exp

#31. 'chemotherapy'/exp

#30. 'cancer chemotherapy'/exp

#29. #13 AND #28

#28. #14 OR #15 OR #16 OR #17 OR #18 OR #19 OR #20 OR #21 OR #22 OR #23 OR #24 OR #25 OR #26 OR #27

#27. 'mix*':ab,kw,ti

#26. 'differentiation*':ab,kw,ti

#25. 'difference*':ab,kw,ti

#24. 'variant*':ab,kw,ti

#23. 'nonpure':ab,kw,ti

#22. 'small cell':ab,kw,ti

#21. 'adenocarcinom*':ab,kw,ti

#20. 'squamous':ab,kw,ti

#19. 'lymphoepithelioma-like':ab,kw,ti

#18. 'micropapillary':ab,kw,ti

#17. 'sarcomatoid carcinoma'/exp

#16. 'small cell carcinoma'/exp

#15. 'adenocarcinoma'/exp

#14. 'squamous cell carcinoma'/exp

#13. #1 OR #12

#12. #7 AND #11

#11. #8 OR #9 OR #10

#10. 'transitional cell':ab,kw,ti

#9. 'bladder':ab,kw,ti

#8. 'urothel*':ab,kw,ti

#7. #2 OR #3 OR #4 OR #5 OR #6

#6. 'neoplasm*':ab,kw,ti

#5. 'papillom*':ab,kw,ti

#4. 'tumor*':ab,kw,ti

#3. 'carcinom*':ab,kw,ti

#2. 'cancer*':ab,kw,ti

#1. 'bladder cancer'/exp

Database:cochrane < to 2021 Dec 01 > 62 Search Strategy:

#1 MeSH descriptor: [Urinary Bladder Neoplasms] explode all trees

#2 MeSH descriptor: [Carcinoma, Transitional Cell] explode all trees

#3 MeSH descriptor: [Urothelium] explode all trees

#4 (cancer*):ti,ab,kw

#5 (carcinom*):ti,ab,kw

#6 (tumor*):ti,ab,kw

#7 (papillom*):ti,ab,kw

#8 (neoplasm*):ti,ab,kw

#9 #4 OR #5 OR #6 OR #7 OR #8

#10 (urothel*):ti,ab,kw

#11 (bladder):ti,ab,kw

#12 (Transitional Cell):ti,ab,kw

#13 #10 OR #11 OR #12

#14 #9 AND #13

#15 #1 OR #2 OR #3 OR #14

#16 MeSH descriptor: [Carcinoma, Squamous Cell] explode all trees

#17 MeSH descriptor: [Adenocarcinoma] explode all trees

#18 MeSH descriptor: [Carcinoma, Small Cell] explode all trees

#19 (Squamous):ti,ab,kw

#20 (Adenocarcinom*):ti,ab,kw

#21 (Small Cell):ti,ab,kw

#22 (Micropapillary):ti,ab,kw

#23 (Sarcomatoid):ti,ab,kw

#24 (Lymphoepithelioma-Like):ti,ab,kw

#25 (nonpure):ti,ab,kw

#26 (variant*):ti,ab,kw

#27 (difference*):ti,ab,kw

#28 (differentiation*):ti,ab,kw

#29 (MIX*):ti,ab,kw

#30 #16 OR #17 OR #18 OR #19 OR #20 OR #21 OR #22 OR #23 OR #24 OR #25 OR #26 OR #27 OR #28 OR #29

#31 #15 AND #30

#32 MeSH descriptor: [Neoadjuvant Therapy] explode all trees

#33 MeSH descriptor: [Antineoplastic Agents] explode all trees

#34 MeSH descriptor: [Drug Therapy] explode all trees

#35 MeSH descriptor: [Drug Therapy, Combination] explode all trees

#36 MeSH descriptor: [Epirubicin] explode all trees

#37 MeSH descriptor: [Doxorubicin] explode all trees

#38 MeSH descriptor: [Mitomycins] explode all trees

#39 MeSH descriptor: [Mitomycin] explode all trees

#40 MeSH descriptor: [Thiotepa] explode all trees

#41 MeSH descriptor: [Antibiotics, Antineoplastic] explode all trees

#42 (chemo*):ti,ab,kw

#43 (epirubicin):ti,ab,kw

#44 (Doxorubicin):ti,ab,kw

#45 (Adriamycin):ti,ab,kw

#46 (gemcitabine):ti,ab,kw

#47 (Mitomycin*):ti,ab,kw

#48 (Thiotepa):ti,ab,kw

#49 #32 OR #33 OR #34 OR #35 OR #36 OR #37 OR #38 OR #39 OR #40 OR #41 OR #42 OR #43 OR #44 OR #45 OR #46 OR #47 OR #48 227935

#50 #31 AND #49

#51 MeSH descriptor: [Cystectomy] explode all trees

#52 (cystectom*):ti,ab,kw

#53 #51 OR #52

#54 #50 AND #53

#55 MeSH descriptor: [Kaplan-Meier Estimate] explode all trees

#56 MeSH descriptor: [Multivariate Analysis] explode all trees

#57 MeSH descriptor: [Risk Assessment] explode all trees

#58 (Multivaria*):ti,ab,kw

#59 (Cox):ti,ab,kw

#60 (Kaplan Meier):ti,ab,kw

#61 (adjusted):ti,ab,kw

#62 #55 OR #56 OR #57 OR #58 OR #59 OR #60 OR #61

#63 #54 AND #63
