# Supplementary material for: Neoadjuvant and Adjuvant Chemotherapy for Variant Histology Bladder Cancers: A Systematic Review and Meta-Analysis
Source: Front Oncol. 2022 Jul 14;12:907454. doi: 10.3389/fonc.2022.907454 (PMC9333064; doi:10.3389/fonc.2022.907454)
Supplement: Supplementary file 5 [file Table_1.docx]

| **First author** | **Year** | **center** | **Study type** | **Study period** | **VH type** | **NAC regime** | **Factors adjusted for in multivariable analysis** | **Inclusion criteria** | **no.of patients in survival analysis** | **no.of patients received NAC** |
| --- | --- | --- | --- | --- | --- | --- | --- | --- | --- | --- |
| **Gelpi-Hammerschmidt** | **2016** | **NCDB** | **Retrospective** | **1998-2012** | **NE** | **NR** | **comorbidities (not clear)** | **MIBC patients** | **NR** | **2058** |
|  |  |  |  |  | **SA** | **NR** | **comorbidities (not clear)** | **MIBC patients** | **NR** | **679** |
|  |  |  |  |  | **MP** | **NR** | **comorbidities (not clear)** | **MIBC patients** | **NR** | **1397** |
| **Sui** | **2016** | **NCDB** | **Retrospective** | **2004-2014** | **MP** | **NR** | **Age, Sex, Race, Charlson/Deyo score, Insurance, Income, T stage, Grade;** | **≥cT2 disease** | **94** | **31** |
| **Joshi** | **2017** | **NCDB** | **Retrospective** | **2003-2014** | **GL** | **NR** | **age, gender, stage** | **cT2-4N0-3Mx patients** | **494** | **69** |
|  |  |  |  |  | **MP** | **NR** | **age, gender, stage** | **cT2-4N0-3Mx patients** | **270** | **78** |
|  |  |  |  |  | **SQ** | **NR** | **age, gender, stage** | **cT2-4N0-3Mx patients** | **1063** | **128** |
| **Sui** | **2017** | **NCDB** | **Retrospective** | **2004-2014** | **SA** | **NR** | **Age, Sex, Race, Charlson/Deyo score, T stage** | **patients underwent radical cystectomy** | **155** | **35** |
| **Vetterlein** | **2017** | **NCDB** | **Retrospective** | **2003-2012** | **MP** | **NR** | **age, sex, Charlson-Deyo comorbidity index, stage, radiotherapy, and adjuvant chemotherapy** | **≥cT2N0-1M0 patients** | **124** | **27** |
|  |  |  |  |  | **SA** | **NR** | **age, sex, Charlson-Deyo comorbidity index, stage, radiotherapy, and adjuvant chemotherapy** | **≥cT2N0-1M0 patients** | **267** | **38** |
|  |  |  |  |  | **SQ** | **NR** | **age, sex, Charlson-Deyo comorbidity index, stage, radiotherapy, and adjuvant chemotherapy** | **≥cT2N0-1M0 patients** | **697** | **65** |
|  |  |  |  |  | **GL** | **NR** | **age, sex, Charlson-Deyo comorbidity index, stage, radiotherapy, and adjuvant chemotherapy** | **≥cT2N0-1M0 patients** | **327** | **39** |
|  |  |  |  |  | **NE** | **NR** | **age, sex, Charlson-Deyo comorbidity index, stage, radiotherapy, and adjuvant chemotherapy** | **≥cT2N0-1M0 patients** | **231** | **104** |
|  |  |  |  |  | **Other histology** | **NR** | **age, sex, Charlson-Deyo comorbidity index, stage, radiotherapy, and adjuvant chemotherapy** | **≥cT2N0-1M0 patients** | **112** | **29** |
| **Dotson** | **2019** | **NCDB** | **Retrospective** | **2004-2015** | **SQ** | **NR** | **age, Charlson score, T stage** | **cT2-3N0M0 patients** | **604** | **41** |
| **Matulay** | **2019** | **NCDB** | **Retrospective** | **2004-2015** | **SQ** | **NR** | **age, sex, race, type of facility, Charlson comorbidity index, stage, grade** | **cT2-4N0M0** | **1013** | **75** |
| **Stensland** | **2020** | **NCDB** | **Retrospective** | **2004-2013** | **SQ** | **NR** | **age, TNM stage, Charlson comorbidity index, race, sex, and facility and county level variables** | **cT2-3N0M0 patients** | **828** | **53** |
| **Chakiryan** | **2021** | **NCDB** | **Retrospective** | **2004-2017** | **SA** | **NR** | **Age, gender, race, Charlson comorbidity index, facility type, stage** | **cT2-4N0-1M0 patients** | **501** | **106** |
|  |  |  |  |  | **MP** | **NR** | **Age, gender, race, Charlson comorbidity index, facility type, stage** | **cT2-4N0-1M0 patients** | **418** | **135** |
|  |  |  |  |  | **SQ** | **NR** | **Age, gender, race, Charlson comorbidity index, facility type, stage** | **cT2-4N0-1M0 patients** | **1141** | **94** |
|  |  |  |  |  | **NE** | **NR** | **Age, gender, race, Charlson comorbidity index, facility type, stage** | **cT2-4N0-1M0 patients** | **629** | **324** |
|  |  |  |  |  | **GL** | **NR** | **Age, gender, race, Charlson comorbidity index, facility type, stage** | **cT2-4N0-1M0 patients** | **750** | **53** |
| **Diamantopoulos** | **2021** | **SEER** | **Retrospective** | **2004-2015** | **MP** | **(67%) cisplatin-based** | **age, gender,stage** | **patients underwent radical cystectomy** | **63** | **27** |
|  |  | **University of Washington, Seattle Cancer Care Alliance** | **Retrospective** | **2003-2018** | **MP** | **cisplatin-based** | **age, gender, renal function and stage** | **cT2-4 patients** | **37** | **27** |
| **Scosyrev** | **2011** | **Southwestern Oncology Group** | **secondary analysis of RCT** | **1987-1998** | **SQ and GL** | **cisplatin-based** | **age and stage** | **cT2-4aN0M0 patients** | **59** | **32** |
| **El Latif** | **2013** | **Faculty of Medicine, Beni-Suef University** | **Retrospective** | **2004-2011** | **many** | **NR** | **age, smoking, gender, American Society of Anesthesiology score, stage, surgical margins,lymphovascular invasion and carcinoma in situ** | **patients underwent radical cystectomy** | **NR** | **NR** |
| **Lin** | **2013** | **Columbia University College of Physicians and Surgeons** | **Retrospective** | **2000-2012** | **SQ and GL** | **(67%) cisplatin-based** | **Age, Race, Sex, T stage, Positive surgical margin** | **cT2-T4N0-NxM0-Mx** | **33** | **11** |
| **Lynch** | **2013** | **University of Texas MD Anderson Cancer Center** | **Retrospective** | **1985-2010** | **NE** | **(94%) cisplatin-based** | **Gender, stage** | **≤cT4aN0M0** | **95** | **48** |
| **Canvasser** | **2014** | **University of California Davis Medical Center** | **Retrospective** | **1995-2008** | **MP** | **NR** | **age, gender, T stage, Karnofsky Score, and Charlson Index** | **all of MP patienta** | **41** | **12** |
| **Mitra** | **2014** | **University of Southern California** | **Retrospective** | **1976-2008** | **SQ** | **NR** | **age,hydronephrosis, stage,extent of differentiation elements,and neoadjuvant and adjuvant chemotherapy administration** | **patients underwent radical cystectomy** | **141** | **6** |
|  |  |  |  |  | **GL** | **NR** | **age,hydronephrosis, stage,extent of differentiation elements,and neoadjuvant and adjuvant chemotherapy administration** | **patients underwent radical cystectomy** | **97** | **3** |
| **Fernandez** | **2017** | **University of Texas MD Anderson Cancer Center** | **Retrospective** | **1989-2012** | **MP** | **(75%) cisplatin-based** | **Extensive vs focal disease in TUR specimen, Risk group, CIS, Time to Treatment** | **≤cT4aN0M0 patients** | **92** | **NR** |
| **Hajiran** | **2021** | **H. Lee Moffitt Cancer Center and Research Institute** | **Retrospective** | **2007-2017** | **SQ and GL** | **(72%) Cisplatin based** | **age, stage and comorbidities** | **cT2–4N0M0 patients** | **183** | **78** |
|  |  |  |  |  | **except SQ and GL** | **(71%) Cisplatin based** | **age, stage and comorbidities** | **cT2–4N0M0 patients** | **173** | **78** |
| MP, micropapillary; SQ, squamous; GL, glandular; NE, neuroendocrine; SA, sarcomatoid; NR, not reported. | | | | | | | | | | |
