# Supplementary material for: Neoadjuvant and Adjuvant Chemotherapy for Variant Histology Bladder Cancers: A Systematic Review and Meta-Analysis
Source: Front Oncol. 2022 Jul 14;12:907454. doi: 10.3389/fonc.2022.907454 (PMC9333064; doi:10.3389/fonc.2022.907454)
Supplement: Supplementary file 6 [file Table_2.docx]

| **First author** | **Year** | **center** | **Study type** | **Study period** | **VH type** | **AC regime** | **Factors adjusted for in multivariable analysis** | **Inclusion criteria** | **no.of patients in survival analysis** | **no.of patients received AC** |
| --- | --- | --- | --- | --- | --- | --- | --- | --- | --- | --- |
| **Sui** | **2016** | **NCDB** | **Retrospective** | **2004-2014** | **MP** | **NR** | **Age, Sex, Race, Charlson/Deyo score, Insurance, Income, T stage, Grade;** | **≥cT2 disease** | **94** | **NR** |
| **Sui** | **2017** | **NCDB** | **Retrospective** | **2004-2014** | **SA** | **NR** | **Age, Sex, Race, Charlson/Deyo score, T stage** | **patients underwent radical cystectomy** | **155** | **20** |
| **Berg** | **2019** | **NCDB** | **Retrospective** | **2004-2015** | **MP** | **NR** | **age, sex, CCI, surgical margin status, and stage** | **T2-4N+M0/T3-4N0M0 patients** | **157** | **60** |
|  |  |  |  |  | **SA** | **NR** | **age, sex, CCI, surgical margin status, and stage** | **T2-4N+M0/T3-4N0M1 patients** | **287** | **72** |
|  |  |  |  |  | **squamous** | **NR** | **age, sex, CCI, surgical margin status, and stage** | **T2-4N+M0/T3-4N0M2 patients** | **787** | **107** |
|  |  |  |  |  | **GL** | **NR** | **age, sex, CCI, surgical margin status, and stage** | **T2-4N+M0/T3-4N0M3 patients** | **392** | **106** |
|  |  |  |  |  | **NE** | **NR** | **age, sex, CCI, surgical margin status, and stage** | **T2-4N+M0/T3-4N0M4 patients** | **186** | **72** |
| **Deuker** | **2020** | **SEER** | **Retrospective** | **2001-2016** | **MP** | **NR** | **age, T stage and/or N stage** | **T1-2N0M0 patients** | **63** | **27** |
|  |  |  |  |  | **MP** | **NR** | **age, T stage and/or N stage** | **T3–4N0M0/TanyN1–3M0 patients** | **105** | **57** |
|  |  |  |  |  | **MP** | **NR** | **age, T stage and/or N stage** | **Tany Nany M1 patients** | **56** | **38** |
| **Stensland** | **2020** | **NCDB** | **Retrospective** | **2004-2013** | **SQ** | **NR** | **age, TNM stage, Charlson comorbidity index, race, sex, and facility and county level variables** | **cT2-3N0M0 patients** | **828** | **48** |
| **Mitra** | **2014** | **University of Southern California** | **Retrospective** | **1976-2008** | **SQ** | **NR** | **age,hydronephrosis, stage,extent of differentiation elements,and neoadjuvant and adjuvant chemotherapy administration** | **patients underwent radical cystectomy** | **141** | **26** |
|  |  |  |  |  | **GL** | **NR** | **age,hydronephrosis, stage,extent of differentiation elements,and neoadjuvant and adjuvant chemotherapy administration** | **patients underwent radical cystectomy** | **97** | **32** |
| **Kaushik** | **2015** | **University of Texas Health Science Center** | **Retrospective** | **1980-2005** | **NE** | **NR** | **stage,Tumor size,Node positive disease** | **patients underwent radical cystectomy** | **68** | **18** |
| **Zamboni** | **2021** | **multicenter** | **Retrospective** | **1999-2018** | **SQ** | **NR** | **age, gender, CCI, stage, positive surgical margins and neoadjuvant chemotherapy** | **patients underwent radical cystectomy** | **353** | **57** |
|  |  |  |  |  | **SA** | **NR** | **age, gender, CCI, stage, positive surgical margins and neoadjuvant chemotherapy** | **patients underwent radical cystectomy** | **65** | **10** |
|  |  |  |  |  | **MP** | **NR** | **age, gender, CCI, stage, positive surgical margins and neoadjuvant chemotherapy** | **patients underwent radical cystectomy** | **89** | **17** |
|  |  |  |  |  | **GL** | **NR** | **age, gender, CCI, stage, positive surgical margins and neoadjuvant chemotherapy** | **patients underwent radical cystectomy** | **98** | **23** |
|  |  |  |  |  | **NE** | **NR** | **age, gender, CCI, stage, positive surgical margins and neoadjuvant chemotherapy** | **patients underwent radical cystectomy** | **112** | **23** |
| MP, micropapillary; SQ, squamous; GL, glandular; NE, neuroendocrine; SA, sarcomatoid; NR, not reported. | | | | | | | | | | |
